# A Review on Chemical and Autogenous Shrinkage of Cementitious Systems

**DOI:** 10.3390/ma17020283

**Published:** 2024-01-05

**Authors:** Hassan Ghanem, Rawan Ramadan, Jamal Khatib, Adel Elkordi

**Affiliations:** 1 Faculty of Engineering, Beirut Arab University, Beirut 11-5020, Lebanon; r.ramadan@bau.edu.lb (R.R.); j.khatib@bau.edu.lb (J.K.); a.elkordi@bau.edu.lb (A.E.); 2 Faculty of Engineering, University of Wolverhampton, Wolverhampton WV1 1LY, UK; 3 Faculty of Engineering, Alexandria University, Alexandria 5423021, Egypt

**Keywords:** autogenous shrinkage, chemical shrinkage, paste, mortar

## Abstract

Chemical shrinkage (CS) is an intrinsic parameter that may affect the early age cracking of paste, mortar and concrete. It is well known as the driving force of self-desiccation, autogenous shrinkage (AGS) and drying shrinkage. During the first stage of cement hydration (at the initial setting time), the CS and AGS are equal. In the hardened stages, there is a difference in values between the two shrinkage parameters. This paper is a comprehensive review on CS and AGS, measurement techniques, modeling and prediction of different cementitious systems. Based on the various experimental studies, chemical shrinkage depends on the water to binder ratio (w/b) and is proportional to the degree of hydration. A low w/b ratio leads to high CS and AGS. The composition of cement has an effect on both CS and AGS. Also, incorporating supplementary cementitious materials (SCMs) affects both shrinkage parameters. It is concluded that adding fly ash (FA) to concrete contributes to CS and AGS reductions. However, this is not the case when concrete contains slag. More than 170 references were consulted including 35 which were published after 2020. According to the authors knowledge, there is no published work on the effect of fibers, especially bio-fibers, on the chemical shrinkage of cement-based composites. Therefore, in addition to traditional chemical shrinkage of cementitious systems, this review includes a section on recent papers conducted by the authors on the effect of bio-fibers on the chemical shrinkage of cement composites.

## 1. Introduction

The evolution of urbanization in the world led to an increase in the consumption of concrete. Historically, many scientists have succeeded in maintaining a sustainable and eco-friendly concrete material by incorporating supplementary materials into cement [1,2,3,4,5,6,7,8,9,10,11,12,13,14]. These materials play a crucial role in controlling the emission of CO_2_ generated from the cement manufacturing [15,16,17]. Despite these benefits, cracking of concrete remains a critical issue.

An essential shrinkage parameter responsible for theses cracks is CS. It takes place during the hydration of cement, whereas the total hydrate volume created is less than that of its reactants. CS is usually considered as the main driving mechanism, which contributes to early age cracking and durability loss of cement-based materials [18,19]. During the early stages of hydrations and before hardening takes place, the AGS and CS are approximately equal [14,20,21,22]. After that, CS results in developing internal pore structures and capillary internal stresses. In order to determine CS, cement paste must be water-saturated and the external imbibed water needs to replace empty pores, which are responsible for the total volume change.

This paper is a review of the CS and AGS of cementitious materials. It covers the various CS and AGS terminologies, techniques for CS and AGS measurement, correlations between CS and mechanical properties, modeling and predictions of different cementitious systems. A large number (172) of references were consulted to conduct this review, where more than thirty of these references were published in the last three years. Virtually, there is no research on the effect of fibers on the chemical shrinkage of cement-based systems. In addition to the evaluation of CS and AGS of paste and mortar, this review contains one section on the effect of bio-fibers on CS and AGS, which was not reported in previous studies on the topic.

## 2. Chemical Shrinkage and Autogenous Shrinkage Definition

### 2.1. Chemical Shrinkage

The volume of hydrated products of cement paste is smaller than the volume of the reactants (i.e., cement and water). This phenomenon is referred to as chemical shrinkage, hardening shrinkage or Le Chatelier shrinkage [16,17,18,19,23]. Le Chatelier measured the reduction in cement paste volume by means of a simple dilatometer using a glass beaker attached to a tube filled with water. It was found that the total chemical shrinkage (TCS) of Portland cement (PC) was 4.60% by weight of cement. CS is occasionally referred to as “internal autogenous shrinkage” and AGS as “external chemical shrinkage” [24,25]. Additionally, internal autogenous shrinkage (chemical shrinkage) should not be confused with external chemical shrinkage (autogenous shrinkage) which is the change in the external dimensions of materials. In general, the external dimensions can decrease due to self-drying (autogenous shrinkage), external drying (drying shrinkage), or increase due to water absorption (swelling) [26]. Shrinkage will occur even though the hydrated products are smaller than the volume of the reactants (cement and water) and even if the material undertakes external expansion. Moreover, CS is usually up to two times higher than AGS. CS is directly dependent on the degree of hydration.

Additionally, CS depends on the stoichiometry of water. Thus, CS may produce various substitutes of cementitious materials. On the other side, Justnes et al. [27] and Wright et al. [28] defined CS as two measurable quantities: “total chemical shrinkage” (TCS) and “external chemical shrinkage” (ECS) [27,28]. ECS is the change in external cement particle volume due to chemical reactions at early stages. However, TCS is a measure of the paste capacity reaction in the presence of water. TCS is the sum of internal volume changes and external volume changes. However, Reyniers and Vn Loo [29] illustrated that TCS and ECS are equal up to the first 8 h, until the final set. After this period, the ECS mechanism seems to stop once the paste structure shows its rigidity and ability to face the internal contraction forces. However, TCS continues to increase after the paste has hardened (8 h). The difference between TCS and ECS is equal to the volume of the internal contraction pores. Beyond 8 h, TCS continues to increase.

**Figure 1:**
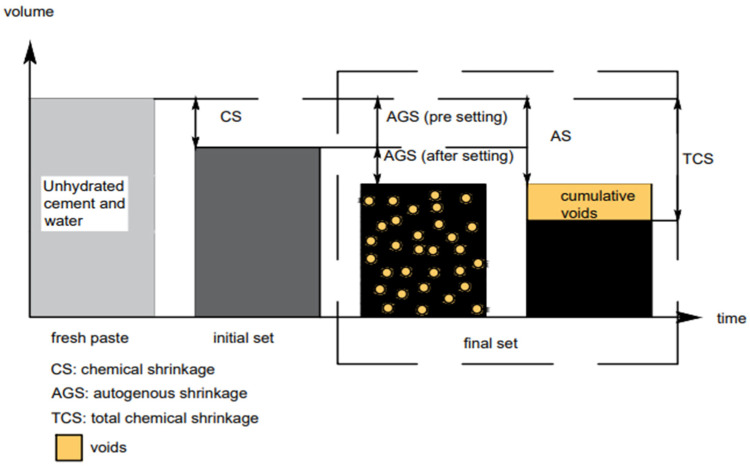
The relation between CS and AGS during hydration phase [27].

**Figure 2:**
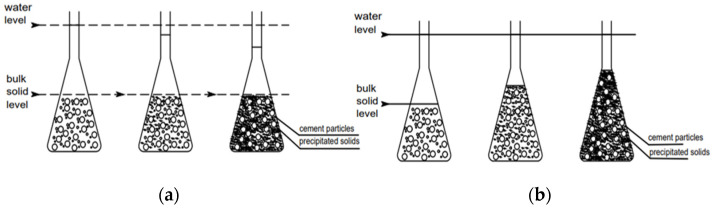
Schematic illustration of (**a**) the total system volume changes and (**b**) the external sample volume changes during hydration stage [30].

The mechanism of CS and AGS is presented in Figure 1. In some circumstances, AGS is negative (expansion), especially in the early stages of hydration and at a high w/c ratio [30]. In addition to that, expansion continues even though the volume of the total system continues to drop. If the total system volume drops (CS) but the specimen external volume increases, then the variation in volume is indicative of an increase in pore spacing [30]. In order to increase the external volume of the paste, the cement particles must expand to form a rigid structure [30]. Figure 2 exhibits a model to show the contrast between CS and external sample expansion. In Figure 2a, the total system volume shows a reduction during the hydration products precipitation phase. Figure 2b displays the stability of the system volume, whenever the external volume of the solid continues to increase. This finding can be due to the restructuring of cement particles in the system such as water absorption and swelling. These two processes occur simultaneously.

### 2.2. Autogenous Shrinkage

As reported by the Japan Concrete Institute (JCI), AGS is the macroscopic volume reduction in cement during early hydration at the initial setting time [19]. Nevertheless, other authors considered autogenous shrinkage as the measured disfiguration of cement paste in a locked system [31,32]. Some research considered the critical effect of AGS in high performance concrete (HPC), where the greater amount of cement and low w/b ratio caused more self-desiccation in the system. Additionally, in concrete with a low w/c ratio (˂0.4), the inner moisture is not enough to completely hydrate the cement particles and encourage the occurrence of AGS [33,34]. Thus, AGS is considered as the critical aspect in early cracking. In fact, most of AGS happens during the first 24 h after the addition of water to the mix. During this stage, the matrix is more prone to cracking due to the low ability of concrete to resist the tensile stresses [35,36].

## 3. Chemical and Autogenous Shrinkage Measurement and Techniques

### 3.1. Chemical Shrinkage Measurement

**Figure 3:**
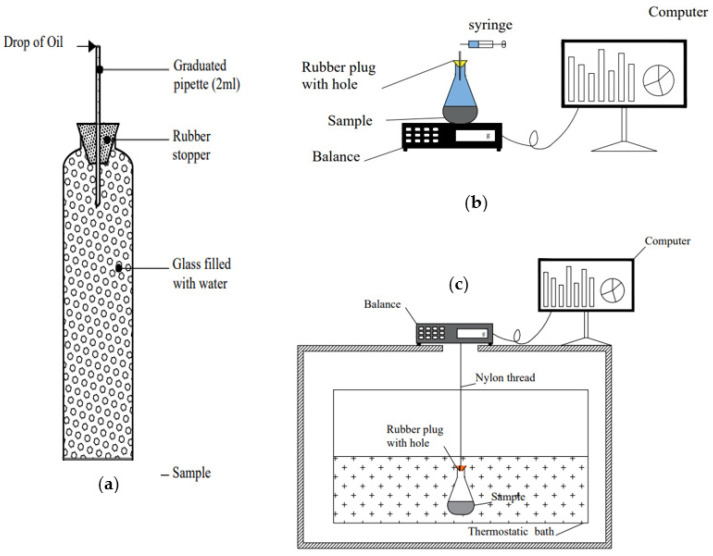
CS measurement techniques: (**a**) Dilatometry [37], (**b**) Pyconometry [58] and (**c**) Gravimetry [62].

CS is measured on a fully saturated sample. Excess water is imbibed into the hydrated cement paste and volume change can be measured. Accordingly, the thickness of the cement paste sample should be small enough to allow water penetration to the entire depth of the sample. The CS can be measured by either the volumetric or gravimetric techniques. The volumetric technique can be conducted using either the dilatometry or the pycnometry method (Figure 3a,b). The first volumetric method involves measuring the change in the water level which is indicative of the volume change inside the sample. This methodology was firstly developed by Le Chatelier [16] and is generally denoted as the dilatometry measurement method [37,38,39] which can be calculated as follows:

(1) $$CS=\frac{\Delta V}{W}$$

where *CS* is the chemical shrinkage of the sample (mL/g of binder), ∆*V* is the volume change in the pipette (mL) and *W* is the weight of the sample $\left(g\right)$.

The second volumetric method is called pyconometry as suggested by Swayze [40]. This method is based on keeping the total volume of paste and water constant, while continuously weighing the sample. It allows data to be recorded automatically. Both methods (dilatometry and pyconometry) have been proposed by ASTM C 1608 [41] and applied in various experimental work [17,24,27,28,42,43,44,45,46,47,48,49,50,51,52,53,54,55,56,57,58]. The third method is the gravimetric method, which was firstly suggested by Rey [59] and developed by several authors [37,60,61,62,63,64,65,66,67]. This method uses Archimedes’ standard in order to estimate the volume changes of hydrated cement paste. Excess water-moistened cement paste is weighed with water or oil (Figure 3c). Consequently, the recorded weight change is related to the CS divided by the liquid density used for weighing. This method which was developed from pyconometry and had many advantages such as the automatic recording of results is also referred to as the buoyancy method and calculated as follows:

(2) $$CS(t,T)=\frac{W_{s}(t)-W_{s}(t_{i})}{W_{c}\rho_{L}(T)}$$

where CS is the chemical shrinkage of the sample (mL/g of binder); $W_{s}\left(t\right)$ is the weight of both the flask and sample at a time $\left(t\right)$; $W_{s}\left(t_{i}\right)$ is the weight of both the flask and sample at the initial time $\left(t_{i}\right)$; *W_C_* is the initial weight of the binder $\left(g\right)$; $\rho_{L}\left(T\right)\;$is the density of liquid inside the reservoir and $\left(T\right)\;$is the liquid temperature $\left({}^{\circ}C\right)$. The *CS* measurement methods are presented in Figure 3.

### 3.2. Autogenous Shrinkage Measurement

AGS has different testing methods depending on whether the sample is in the fresh or hardened stage. The amount of AGS can be conducted using the linear or volumetric technique.

#### 3.2.1. Linear Measurement

**Figure 4:**
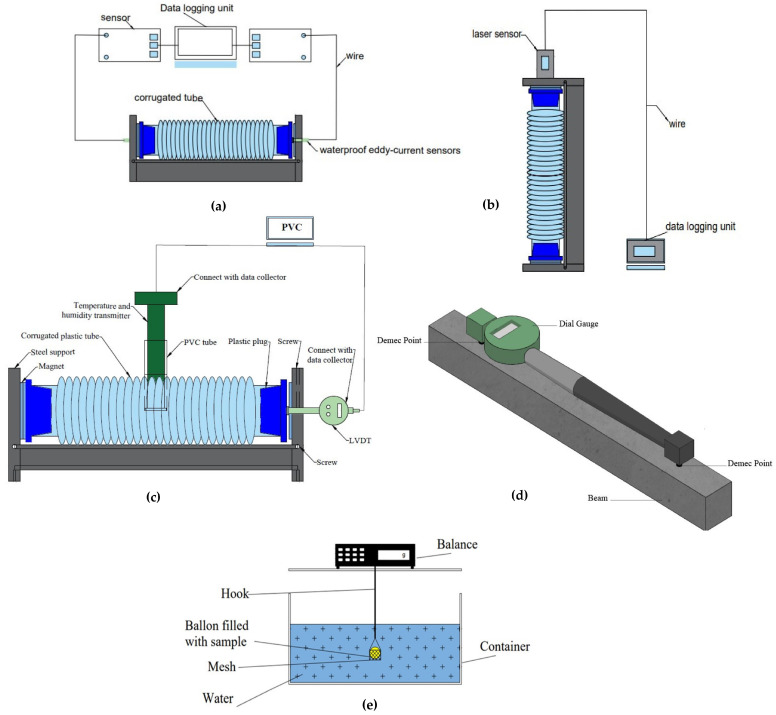
AGS measurements: (**a**) corrugated plastic tube (horizontal direction [57]); (**b**) corrugated plastic tube (vertical direction [71]); (**c**) new device for linear horizontal measurement [72]; (**d**) linear autogenous shrinkage measurement in hardened stage [73]; (**e**) volumetric autogenous measurement device [56].

The cementitious matrices’ autogenous deformations are measured in both horizontal and vertical directions. The experimental system is made up of a flexible, ringed, molded polyvinyl chloride (PVC) membrane that is filled with fresh cement paste or mortar. Care is taken to prevent air bubbles during casting. The membrane is then sealed with metallic caps and placed on a rigid PVC frame. For the “horizontal device”, two waterproof non-contacting displacement sensors are positioned at each end of the frame, operating on the eddy current principle [68,69]. This waterproof characteristic is crucial as it allows the experimental device to remain underwater and the sample to be kept in quasi-isothermal conditions as shown in Figure 4a. For the “vertical test” device, a laser sensor located at the top of the experimental system measures the length changes. This sensor is not waterproof, and only the lower part of the frame and the membrane filled with cement-based material are submerged in water during the test as shown in Figure 4b [37,70,71].

Recently, Zhang et al. [57] have improved the AGS measurement device based on ASTM C1698-09 [72]. This new device can measure the AGS, the relative humidity (RH), and the internal temperature (IT) during the hydration mechanism. In this method, the paste or mortar is poured into a corrugated plastic tube (Figure 4c), and then, it is tied on a steel support. A magnet, pre-set on one edge of the corrugated plastic tube plug, is installed to one end of the steel support, and the second plug is left without setting restrictions so that it could move liberally. The length change of the samples is measured by LVDT. The humidity and temperature transmitter (±0.8% RH) enclosed with a PVC tube is inserted in the center of the sample to measure the temperature and relative humidity. The temperature and humidity transmitters, as well as the LVDT, were connected to a computer. The length change, RH and internal temperature data are collected every 10–15 min. On the other hand, the other main methods involve the use of dial gauge apparatus. These methods are used to measure samples in the hardened stage [73]. According to ASTM C157 [74], the samples are cast in steel molds for 24 h. After this period, two demec points at a distance of 200 mm are applied. These samples are weighted, recorded and placed in plastic bags to prevent any loss in moisture. The length changes of these specimens are then recorded every 2 days by using a dial gauge (Figure 4d).

#### 3.2.2. Volumetric Measurement

The most popular volumetric measurement for AGS is the buoyancy method (or membrane method). This method is built on volume sample changes in a liquid. The elastic membrane bag is filled with a fresh sample, sealed and submerged immediately in a liquid. AGS is evaluated by recording the volume change of the elastic membrane weight over time [56]. Some authors found that volumetric autogenous shrinkage measurement is significantly greater than linear autogenous shrinkage measurements [75]. Justnes et al. [42] investigated the volumetric shrinkage method. The device used for measuring early autogenous shrinkage is presented in Figure 4e. After mixing, the sample is poured inside a flexible membrane (e.g., balloon). In order to remove the air bubbles from the samples, the balloon is gently vibrated for one minute. After that, the balloon was tied, measured and placed on a balance to record the initial dry weight. The sample was then submerged in water and hung under a digital balance using a rod. Then, the sample was continuously recorded every hour. The change in weight represents the volume change of the sample (i.e., water displaced). The volume change can be converted to linear change using the following formula [30]:

(3) $$\frac{\Delta V}{V}=\frac{3\Delta L}{L}$$

where ΔV is the volume change in the pipette, V is the original volume of the paste, ΔL is the length change and L is the original length between two demec points. The records are conveyed in µm/m.

#### 3.2.3. Restrained Deformations: Quasi-Isothermal Ring Test Device

**Figure 5:**
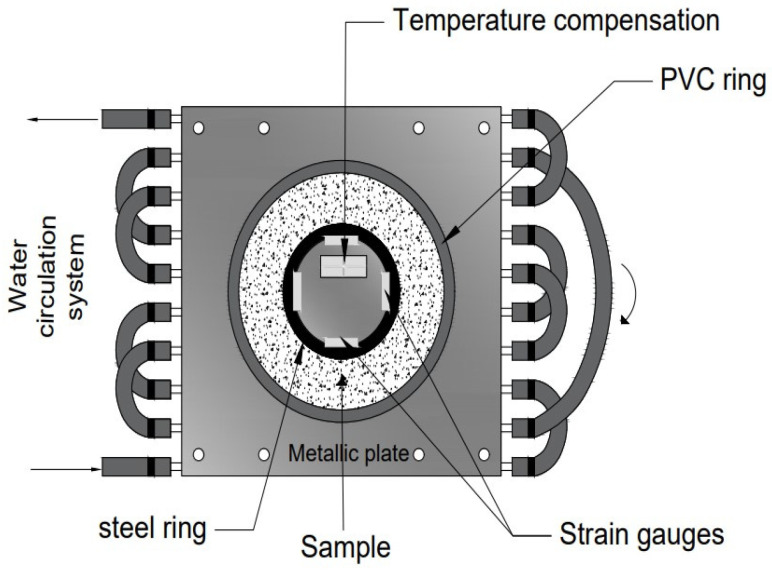
Quasi-isothermal ring test device [75].

To measure the deformations in restrained conditions, the “ring test” method is used, which involves casting fresh cement-based material around a steel ring equipped with strain gauges connected to a data acquisition system [75]. To ensure quasi-isothermal conditions during the tests, a peripheral water circulation system is used with the temperature controlled by a thermostatic water bath. The device automatically monitors the deformations of the steel ring and detects the moment when the cement-based material cracks. Figure 5 provides the ring test device system.

## 4. Factors Affecting Chemical and Autogenous Shrinkage

### 4.1. Cement Composition

**Figure 6:**
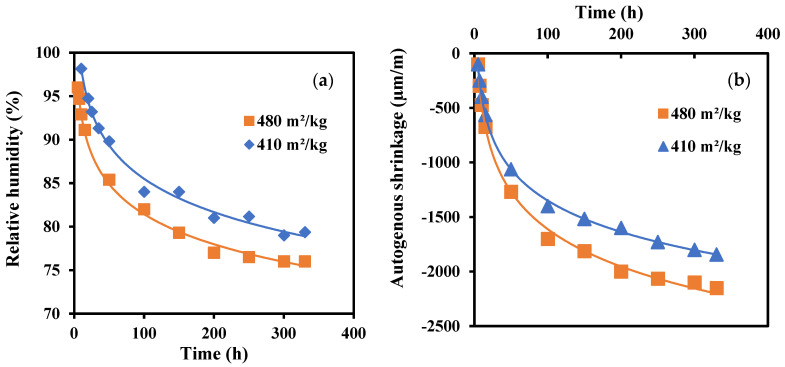
Effect of cement fineness on (**a**) relative humidity and (**b**) autogenous shrinkage [84].

Cement is an essential component in cement-based materials. Its clinker composition consists of tricalcium silicate (C_3_S), tricalcium aluminate (C_3_A), dicalcium silicate (C_2_S) and tetracalcium aluminate ferrite (C_4_AF). Obviously, the concrete structure is liable to producing cracks, especially in the early stages of hydration. This is mainly due to the heat of the hydration degree of C_3_S and C_3_A. Later, the volume reduction in hydration products generated by the chemical reaction of cement composition with water and the consumption of water by the internal pores is referred to as chemical shrinkage [17,19,76]. Generally, the hydration of C_3_A is faster than that of C_2_S, C_3_S, and C_4_AF [77]. This would promote a large difference in the internal and external temperature of concrete. Thus, this leads to an increase in the CS and AGS of concrete [78,79]. Additionally, due to the difference in the chemical and physical composition, the type of cement affects the CS and the AGS. For example, cement with high content of C_3_A and C_3_S responsible for high, early strength cement has the ability to cause more CS than cement with a high content of C_2_S (low heat cement type). A study conducted by Bullard et al. [80] showed that an increase in cement fineness reduces the RH of the cementitious system (Figure 6a), thus increasing the AGS. This may be due to the accelerated hydration of cement that leads to water consumption in the matrix, which also enhances the internal capillary pressure as well as the AGS deformation. On the other hand, PC with high fineness particles leads to a rapid reaction rate, resulting in a further contraction phase of CSH gel. In the first 72 h (3 days), it was stated that most of the AGS occurs. Beyond that age, the rate of hydration gradually dropped, and the autogenous shrinkage of the cement leveled off (Figure 6b). Consequently, the fineness of the cement could contribute to a reduction in the AGS [81,82,83].

Another study by Malhotra [84] investigated the use of coarsely ground cement (particles larger than 40 µm) on AGS in HPC. It was found that the addition of coarsely ground cement in HPC absorbs water for a long period of time and consequently increases the long-time rate of hydration and reduces the AGS. Moreover, cement morphology should be emphasized. For example, spherical cement particles have a larger radius and smaller contact area, which is very efficient in decreasing the AGS of concrete [85].

### 4.2. Supplementary Cementitious Materials

Supplementary cementitious materials (SCMs) are becoming very important in concrete production as they may improve the mechanical and durability properties. Also, due to the reduction in cement content, the use of SCM results in a reduction in CO_2_ emissions from cement manufacturing. Additionally, SCM can affect concrete shrinkage types [86,87,88,89,90,91,92,93,94,95,96]. For example, the rate of AGS at an early age of hydration is affected by the content of silica fume (SF). Jensen et al. [72] reported that at 28 days of curing, the AGS increased by as much as three times when cement is replaced with 10% silica fume at a water binder ratio of 0.3. Similar results were observed by Zhang et al. [97].

Another study highlighted the effect of incorporating 5% and 10% of SF on the AGS of two different w/c ratios (0.26 and 0.35). It was observed that AGS increased with the increase in SF content, especially when a low water cement ratio was used [98]. At a 0.26 w/c ratio, the AGS values of SF concrete samples were more than 100 µɛ at 2 days. This can be explained by the fact that the presence of SF causes pore refinement [98,99]. These findings were in agreement with the results reported elsewhere [100,101,102]. Additionally, SF due to its large surface area can accelerate the cement hydration. The large surface area provokes a rapid reaction of SF and mixing water. Thus, improving the pore space in paste drops the IH of the matrix and intensifies the self-desiccation process [98,99,103,104].

Tazawa et al. [76] reported their findings on AGS on concrete containing slag for surface areas greater than 400 m^2^/kg. They found that the AGS of concrete was proportional to the quantity of slag for up to 75% cement replacement. Beyond that level, the AGS started to drop. They also found that as the surface area of slag particles increased up to 836 m^2^/kg, the AGS decreased by up to 60%. Generally, the influence of slag fineness on CS and AGS originates from the worthy impact of fineness on its reactivity with cement. Another study by Lee et al. [105] focused on the effect of adding 30% and 50% of slag as a cement replacement on the AGS with two w/b ratios (0.27 and 0.32). It was stated that AGS increased as w/b went down and increased as the slag content went up. For example, the AGS strain for 50% replacement of slag reached a value of 410 × 10^−6^ µɛ and 490 × 10^−6^ µɛ when 0.32 w/b and 0.27 w/b were used, respectively. However, the addition of 30% slag yielded an AGS value of 380 × 10^−6^ and 450 × 10^−6^ at w/b of 0.32 and 0.27, respectively [105]. The higher AGS was attributed to the greater CS, which act as a driving force in the concrete with slag. However, as reported previously, slag is more efficient on AGS than SF and FA [106]. This is due to the higher volume of coarse pores in slag which causes a decline in capillary pore pressure and hence reduces the AGS [90]. Nonetheless, other studies disputed the increasing influence of GBFS on the AGS and expansion of concrete [107,108].

Among other SCMs, FA is the most effective mineral admixture controlling the CS and minimizing the AGS. A study by Malhotra, [84] showed that FA had a noticeable effect on the AGS of concrete due to the reduction in IH in the system. It was noticed that the inclusion of more than 60% FA preserved the IH of concrete for up to 7 days. In addition, another study found that the inclusion of more than 50% FA substantially dropped the AGS of the concrete sample having a low w/b ratio of 0.3 [109]. AGS is related to two hydration processes: firstly, it is related to the hydration of PC with Al_2_O_3_ to create ettringite and secondly to the hydration of FA; the latter carried out the decline of the shrinkage process. Moreover, the incorporation of up to 20% FA contributed to reducing the AGS by half, while adding 40% of it led to a sharp reduction in shrinkage compared to plain samples [110]. The increased content and fineness of FA further declined the AGS of concrete as well as prolonging the initial time of cracking. This can be elucidated by the fact that the slow hydration and dilution effect of fly ash as compared to cement contributes to a reduction in the early stage of shrinkage. On the other hand, incorporating FA in a small amount cannot predictably decline the AGS. A study investigated by Jiang et al. [111] showed a remarkable reduction in AGS with the incorporation of a small amount of FA (10%).

Additionally, it should be noted that early crack formation is not preventable by including FA alone [112,113]. Nowadays, nano-materials have been widely used due to their advantages of enhancing early strength and durability. In the early stage, it was shown that the AGS and CS of paste increased with an increase in nano-silica. The addition of 1.2% of nano-silica increased the CS by 57.5% [114].

**Figure 7:**
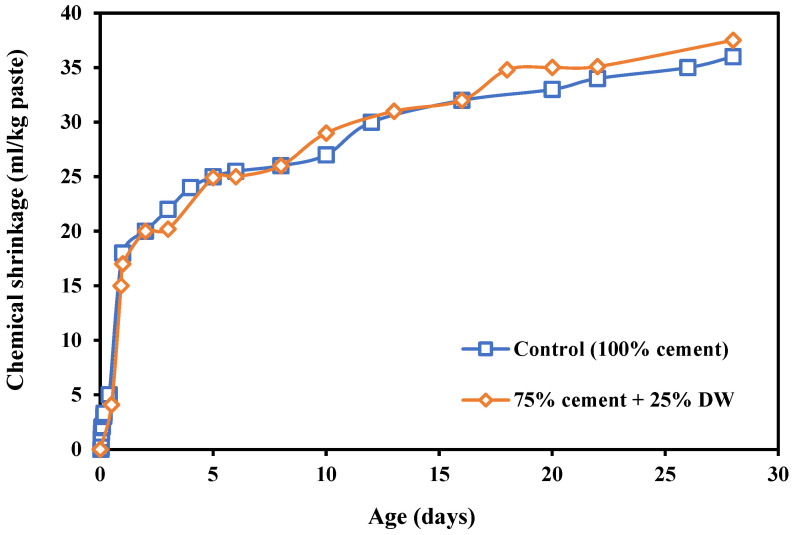
TCS of different paste samples with sulphate-based materials [28].

Wright et al. [28] reported the effect of incorporating desulphurized waste (DW) on CS. The DW is mainly FA with a small quantity of gypsum. The DW caused a delay in the early C_3_A and C_3_S hydration [115,116]. Results are presented in Figure 7. As shown, there was a small increase in CS due to the presence of DW. This can be due to the pozzolanic reactions of FA, which contribute to the hydration progress by altering the type or amount of the hydrates and lead to encouraging the CS. The hardened material density could change if a change in the hydration product took place. Therefore, by incorporating a pozzolanic material (FA), the number of CH created will reduce, and simultaneously, a new C-S-H gel is formed. Thus, the density of the paste will definitely go up [117]. The estimation of FA-G blends displayed that TCS was similar for all mixes during the first 12 h [23,118,119]. TCS started to decrease during the first 7 days of hydration. This is due to the remarkable increase in the skeleton content in the FA-G blends. After this period, the increase in the gypsum continues to improve the TCS for gypsum contents of 25% SO_3_. Many authors stated that the activation of FA could be enhanced in the existence of calcium sulphate, which leads to an improvement in the physical and mechanical properties of cement [28,120,121].

**Figure 8:**
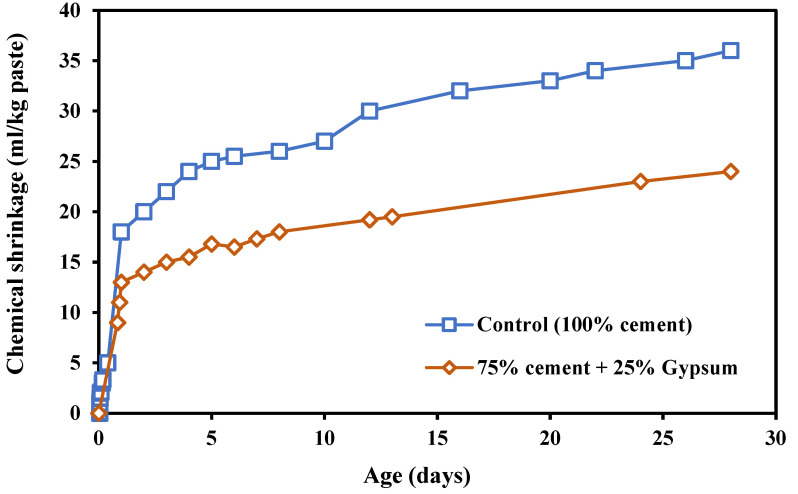
Chemical shrinkage of paste with calcium sulphate based-materials [28].

Wright et al. [28]; Navarrete et al. [122]; Telesca et al. [123]; Liu et al. [124]; and Ragipani et al. [125] examined the effect of DW on the CS and AGS of concrete. It was noted that, during the first 12 h, there was a sharp drop in CS due to the incorporation of calcium sulphate–based materials (CaSO_4_). This last finding indicates that the incorporation of CaSO_4_ delays the creation of hydrates, which leads to a decline in CS [123,125]. ECS in mixes with mineral additives was higher than those of the control mixes. This has caused an increase in the setting time and dropped the degree of hardening. In general, the ECS continues to increase until achieving a sufficient paste strength capable of withstanding the internal contraction forces. This can be explained by the fact that, if the hydration cement process is delayed, the hydrates may not form which result in a lower ECS. On the other hand, a huge reduction in TCS could take place with the incorporation of calcium-based materials. As shown in Figure 8, a large decrease in TCS occurred due to the substitution of cement with calcium sulfate which could be due to the hydration delay caused by the presence of a higher quantity of gypsum. Additionally, it has been illustrated that mixes with SiO_1_-Al_2_O_3_-CaSO_4_-based desulphurized wastes showed an increase in the ECS. This finding requires an acceleration of the early cement hydration reaction, which enhances the quantity of hydrates [122,123,124,125]. Furthermore, the ECS seems to stabilize between 12 and 24 h.

**Figure 9:**
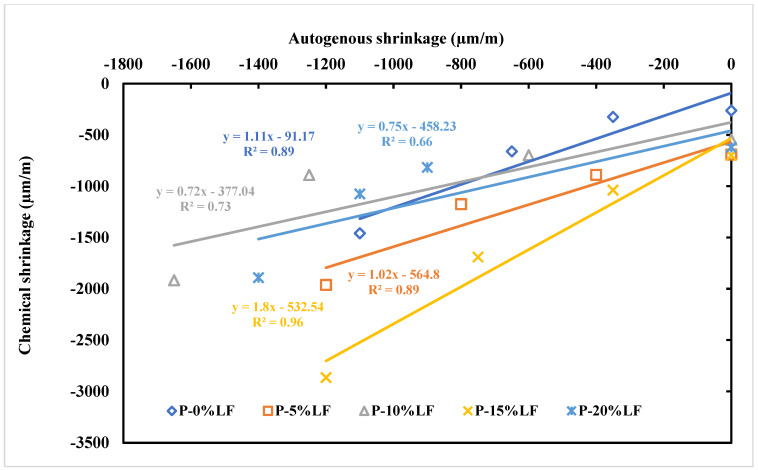
Relation between shrinkage parameters for paste samples [130]. P = paste.

**Figure 10:**
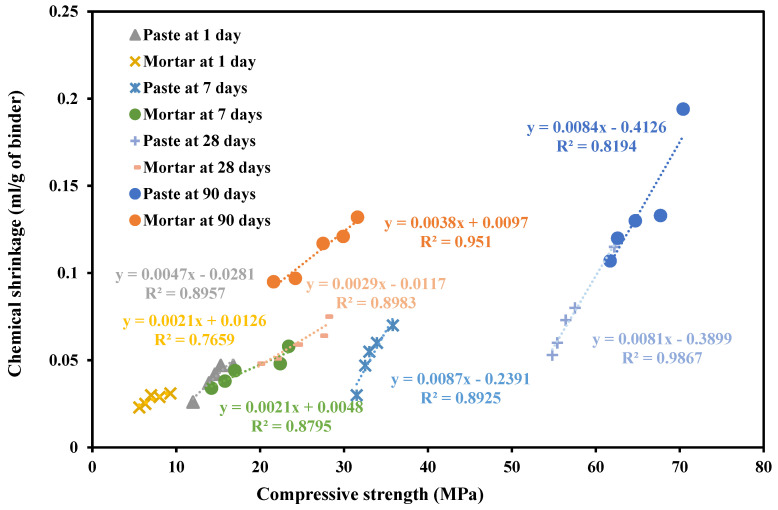
Correlation between compressive strength and chemical shrinkage for pastes and mortars at different curing ages [133].

Many authors have examined the effect of incorporating limestone fines (LF) as a cement replacement on the chemical and autogenous shrinkage of cementitious systems. The presence of LF in cement-based mixes stimulated the growth of CS [126,127,128]. An experimental study concluded that mixing LF with calcined clay (CC) substantially increased the chemical shrinkage during the first 7 days [129]. Khatib et al. [130] found that adding between 10 and 15% of LF increased both the AGS and CS of paste. Additionally, it was reported that a high correlation existed between the two shrinkage parameters as shown in Figure 9. Also, Khatib et al. [131] and Khatib et al. [132] examined the CS and AGS of mortars for a total period of 90 days and stated that adding up to 10% LF increased both shrinkage parameters. Another study conducted by Khatib et al. [133] focused on the correlation between CS and compressive strength of pastes and mortars at different percentages of LF (0 to 20%) and different curing ages (1 to 90 days). As shown in Figure 10, there exists a correlation between CS and compressive strength. The finding indicates that CS is an essential parameter in predicting the development of compressive strength. The presence of LF accelerates the CS and hydration mechanism at early ages [134,135]. Contrary, Wang et al. [136] reported that LF reduced the rate of CS and increased the humidity of the matrix which might be caused by the presence of LF filling the internal pores in the paste. A study conducted by Courard and Michel [137] reported that adding a small amount of LF enhanced the AGS of concrete. However, the addition of LF with a particle size of 16 µm as the cement substitution decreased the AGS of concrete [138].

Metakaolin has a noticeable impact on the CS and AGS of paste. Wild et al. [28] concluded that using up to 15% of MK enhanced both the CS and AGS. This can be due to (1) the reaction between AS_2_ in MK and CH in cement leading to the production hydrate compounds such as C-S-H gel, C_2_ASH_8_ and C_4_AH_13_ or (2) the convergence of low density C_2_ASH_8_ into a high density C_4_AH_13._ These results are in agreement with those reported by Markandeya et al. [139].

### 4.3. Degree of Hydration

**Figure 11:**
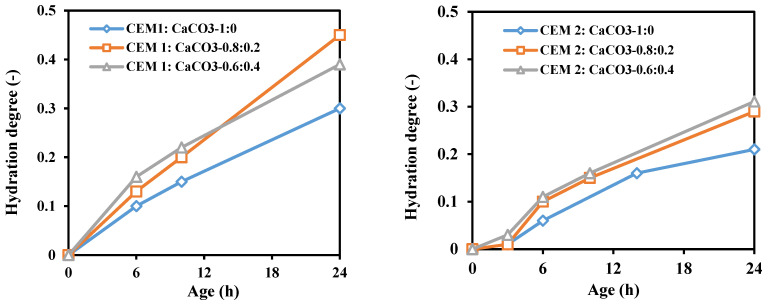
Hydration degree of CEMI-a and CEMII of cement paste at a w/c of 0.4 [143].

Generally, the degree of hydration is defined as the part of cement that has been completely reacted with water relative to the total cement content in the paste. Moreover, the degree of hydration can be affected by w/b, cement type, SCMs and temperature. The hydration degree is modeled by applying diverse techniques like microstructural analysis or thermodynamics [140,141,142]. As reported by Chen et al. [143], the model of CS and AGS is suitable during the first week of hydration. The modeling is achieved using the stratifying capillary tension method. The progression of the hydration degree and the saturation fraction are calculated by applying calometric data and a power volumetric model, respectively. Bouasker et al. [134] reported the evolution of the hydration degree of two types of cement (CEMI and CEMII). It was stated that LF encouraged the initial hydration rate regardless of the types of cement. This can be explained by the presence of LF particles which may act as nucleation sites leading to an increase in the hydration reaction. During the first 12 h, the increase in the LF content (from 20 to 40%) had not caused further acceleration of the cement hydration. However, beyond this period of time, the hydration rate for CEMII is greater than that for CEMI-a as shown in Figure 11.

### 4.4. Water to Binder Ratio

The effect of water binder ratio on CS and AGS has been a point of controversy among researchers [17,27,37,42,63,64,134,144,145]. Some research [65,66,108] indicated that CS has no effect on the w/b ratio and degree of hydration. However, other researchers indicated that CS increased with the increase in the w/b ratio [26,42,46,146,147]. Concrete with a low w/b ratio (˂0.4) has more effect on AGS [97,148,149]. Zhang et al. [97] studied the effect of using different w/b ranging from 0.3 to 0.35 on HPC with and without SF. Results indicated that the AGS increased with the decline of the w/b for the control mix. However, in the presence of SF, the influence of the w/b ratio on the AGS was less noticeable. Jiang et al. [36] examined the role of the w/b ratio, ranging from 0.2 to 0.45, on the AGS for concrete containing SF and slag. It was noted that the inclusion of slag (from 20 to 40%) and SF (from 20 to 40%) to the mix decreased the AGS during early ages of hydration (first two weeks). It was shown that this reduction is more pronounced at a w/b of 0.25. Another study conducted by Wang et al. [128] used a segmental screw micrometer to determine the AGS of cementitious materials for ultra-low w/b. Results showed that the relation between AGS and w/b is inversely proportional when the w/b is above 0.25. This was attributed to the pore structures and low heat of hydration of cementitious materials.

### 4.5. Chemical Admixtures

**Figure 12:**
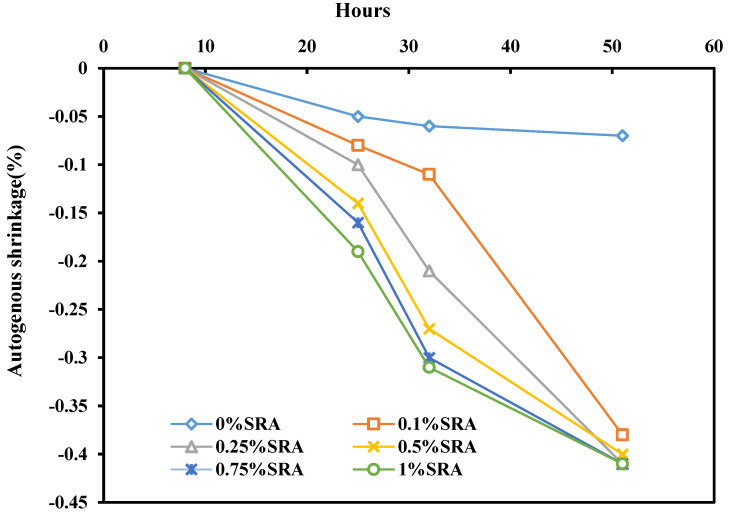
Percentage decrease in autogenous shrinkage with different percentages of SRA [156].

Chemical admixtures (CA) are becoming essential for concrete production. They consist of organic or inorganic chemical molecules and are used to enhance concrete properties. Many authors studied the effect of incorporating CA on the AGS of concrete [150,151,152,153]. For example, Rongbing and Jian, 2005, reported that the addition of 2% shrinkage reducing agent (SRA) by weight of cement contributed to a reduction in the AGS of mortar by up to 60% in the first 60 days and a further reduction of up of 30% at 90 days of curing. This finding indicates that SRA has an important role in delaying the capillary pressure development and reducing cracks during the plastic stage [154]. Another study conducted by Yoo et al. [155] revealed that the AGS of concrete decreased by 18% and 34% when 0.5% and 1% of SRA were used, respectively, compared to the control mix. Kotrla et al. [156] studied the effect of SRA on the AGS of alkali-activated materials and found that the inclusion of 0.1% and 0.25% of SRA caused a 20% reduction in the AGS of concrete, while the addition of 0.5% and 0.75% of SRA reduced it by half compared to the control mix (Figure 12). Also, it can be shown that the incorporation of 1% SRA had an efficient role in reducing the AGS of concrete by 87%. This reduction was related to the synergistic effect of chemical agents. In addition, Saliba et al. [157] studied the influence of incorporating SRA on AGS with the w/b ratio of 0.43 and 0.65. It was observed that the AGS was reduced by only 14 and 20% for w/b ratios of 0.43 and 0.65, respectively. Malhotra et al. [84] reported the combined effect of RA and expansive agent (EA) on the AGS of HPC concrete containing 10% SF. A remarkable decrease of 50% in AGS was observed when EA and SRA were combined. Moreover, Park et al. [158] focused on the influence of CA on the AGS of ultra-high performance of concrete (UHPC). Results showed that the addition of 7.5% of EA and 1% of SRA contributed to reducing the AGS by 24% compared to the control mix.

### 4.6. Effect of Bio-Fibers on CS and AGS

Recent studies examined the effect of incorporating bio-fibers on various shrinkage parameters, with a specific focus on autogenous and chemical shrinkage [159,160,161]. The bio-fibers were obtained from the Phragmites australis plant (PA). The fibers had a length of 20 mm, a width of 2 mm and a thickness of about 1 mm. They were then dried and treated in a 4% sodium hydroxide solution. The PA fiber addition was 0, 0.5, 1, and 2% by volume of cementitious materials. It was observed that adding fibers caused a noticeable reduction in CS and AGS for both paste and mortar. The reduction in CS and AGS was 21.4% and 30.2% for paste, respectively, whereas the associated reduction for mortar was 30.2% and 54% at 2% fiber [159,160].

## 5. Modeling and Prediction

### 5.1. Chemical Shrinkage

Several studies attempted to model analytically the growth of cement hydration [162,163,164,165]. Generally, the semiempirical model are used which consists of the various chemical equations due to cement hydration. The production of CH in cementitious systems is directly related to the internal volume change of the hydration products. Calculating the chemical shrinkage (Δ*V*) and the CH amount produced by the hydration reactions is possible by knowing the stoichiometry equation and the density of each reactant and product. Furthermore, the hydration kinetics equations of all cement compounds should be known in order to determine the progression of both parameters as a function of cement hydration degree and time [166,167]. Generally, PC is a multi-mineral compound and has a complex hydration. A set of simplified equations were described to show the hydration of PC as follows [26].

(4) $$C_{3}S+5.3H\to C_{1.7}SH_{4}+1.3CH$$

(5) $$C_{2}S+4.3H\to C_{1.7}SH_{4}+0.3CH$$

(6) $$C_{3}A+3C\bar{S}H_{2}+26H\to C_{3}A\cdot 3C\bar{S}\cdot H_{32}$$

(7) $$2C_{3}A+C_{3}A\cdot 3C\bar{S}\cdot H_{32}+4H\to 3\left[C_{3}A\cdot C\bar{S}\cdot H_{12}\right]$$

(8) $$C_{3}A+CH+12H\to C_{4}AH_{13}$$

(9) $$C_{4}AF+3C\bar{S}H_{2}+27H\to C_{3}\left(AF\right)\cdot 3C\bar{S}\cdot H_{32}+CH$$

(10) $$2C_{4}AF+C_{3}\left(AF\right)\cdot 3C\bar{S}\cdot H_{32}+6H\to 3\left[C_{3}\left(AF\right)\cdot C\bar{S}\cdot H_{12}\right]+2CH$$

(11) $$C_{4}AF+2CH+10H\to 2C_{3}\left(AF\right)H_{6}$$

(12) $$1.1CH+S+2.8H\to C_{1.1}SH_{3.9}$$

Equations (4)–(11) represent the hydration reactions of the major PC minerals (alite C_3_S, belite C_2_S, tricalcium aluminate C_3_A and ferrite C_4_AF), whereas Equation (12) represents the pozzolanic reaction. Equations (4) and (5) correspond to the hydration of C_2_S and C_3_S. This hydration produces a calcium silicate hydrate (C-S-H) gel in the form of C_1.7_SH_4_ and CH which nucleate and mature in the unfilled capillary pores [168]. Equations (6)–(8) are concerned with the hydration reaction of C_3_A. Equation (9) describes the reaction of C_3_A with gypsum which leads to the formation of ettringite. If the sulfate produced from the gypsum is entirely consumed before the total hydration of C_3_A, ettringite is then converted to mono-sulfoaluminate, as presented in Equation (7). After the transformation of the ettringite, the residual C_3_A reacts with CH as shown in Equation (8). The reactions of the ferrite (C_4_AF) are presented in Equations (9)–(11). The hydration of C_4_AF leads to the formation of $C_{3}\left(AF\right)\cdot 3C\bar{S}\cdot H_{32}$ (Equation (9)) in addition to CH, which is formed due to the higher molar ratio (Ca/Al). The residual C_4_AF reacts with $C_{3}\left(AF\right)\cdot 3C\bar{S}\cdot H_{32}$ to form $3\left[C_{3}(AF)\cdot C\bar{S}\cdot H_{12}\right]$ (Equation (10)). Also, C_4_AF reacts with CH to form $2C_{3}\left(AF\right)H_{6}$. All of these chemical reactions mentioned above are distinguished by the main difference between the reactant and product volumes. Each reaction is unique in having its own stoichiometry which results in different CS values. The values of this chemical reaction are calculated using the density and stoichiometry reaction of each element. For example, the CS of alite can be calculated via dividing the molar mass by the density. The details of calculation are mentioned below [168].

**C_3_S****+****5.3 H****→****C_1.7_SH_4_****+****1.3 CH**Molar volume (V_m_), cm^3^/mole71.13
18.07
107.82
33.08Density (ƿ), g/cm^3^3.210
0.997
2.110
2.240Molar mass (M_r_), g/mole228.330
18.016
227.490
74.096
71.13 cm^3^ + 95.4 cm^3^ 166.53 cm^3^→107.82 cm^3^+ 43.00 cm^3^ 150.82 cm^3^Chemical shrinkage= −15.71 cm^3^/mole of C_3_S hydrated
= −0.069 cm^3^/g of C_3_S hydrated

Generally, the total volume of hydrates is less than the volume of the initial reactants. This difference in volume is referred to as CS. However, specific chemical shrinkage ($\bar{CS}$) is the CS per unit weight of hydrates. The CS $\left(\Delta V_{cs}^{i}\right)$ at any time $\left(t\right)$ can be calculated as the product of a given CS with a precise mineral $\left(CS_{i}\right)$, and the mineral percentage in Cement $\left(i\right)$, the original cement weight $\left(C\right)$ and the hydration degree of the mineral $\left(\alpha_{i}\right)$ are as presented in Equation (13) [164].

(13) $$\Delta V_{cs}^{i}(t)=CS_{i}\times \left[i\right]\times C\times \alpha_{i}(t)$$

The TCS is the sum of the CS of its component minerals as shown in Equation (14). To use Equations (13) and (14) on a regular basis, a multi-mineral hydration model capable of predicting the degree of hydration of individual minerals is required [161]. Such models are generally very complex. Therefore, it is common to simplify Equations (13) and (14) by the average degree of hydration ($\bar{\alpha_{i}}(t)$) and the average $\bar{CS}$ as shown in Equation (15) [164].

(14) $$\Delta V_{CS}^{total}(t)=\sum_{i}\Delta V_{CS}^{i}(t)$$

(15) $$\Delta V_{CS}^{total}(t)=C\times \bar{\alpha }(t)\times \sum_{i}(CS_{i}\times \left[i\right])=\;C\times \bar{\alpha }(t)\times \bar{C}\bar{S}$$

Equation (15) has been used by some researchers to determine the TCS, which is independent of the w/c [81,169]. For this reason, other researchers determine CS using other techniques which include chemically bound water [146,164], heat of hydration [147,158] and compressive strength [170].

### 5.2. Autogenous Shrinkage

The prediction of AGS is important for determining the extent of cracks; thus, predicting concrete long-term durability. The development of AGS depends on the w/b ratio. According to previous experimental data, JCI suggested a prediction model in order to determine the AGS of concrete [17]. The equations are as follows:

(16) $$\begin{array}{l} \varepsilon_{c}(t)=\gamma \times \varepsilon_{c_{0}}\left(\frac{W}{b}\right)\times \beta (t)\times 10^{-6} \end{array}$$

where

(17) $$\begin{array}{c} For:\;0.2\leq \frac{w}{b}\leq 0.5\to \;\varepsilon_{c_{0}}\left(\frac{w}{b}\right)=3070\exp\left[-7.2\left(\frac{w}{b}\right)\right]\\ For:\;\frac{w}{b}>0.5\to \;\varepsilon_{c_{0}}\left(\frac{w}{b}\right)=80\\ \beta (t)=\left[1-\exp\left\{-a{\left(t-t_{0}\right)}^{b}\right\}\right] \end{array}$$

where

$\varepsilon_{c}\left(t\right)$ is the autogenous shrinkage of concrete at time $\left(t\right)$;
γ is a coefficient of cement type (γ=1 for PC);
$\varepsilon_{c_{0}}\left(w/b\right)$ is the ultimate autogenous shrinkage;
$\beta \left(t\right)$ is a coefficient to describe the development of AS with time;
a and b are constants;
t is the age in days;
$t_{0}$ is the initial setting time in days.

Equation (16) is applicable when the concrete is exposed to 20 °C. However, for other temperature, if the temperature is above 20 °C, *t* and *t*_0_ are modified as follows:

(18) $$t,t_{0}=\sum_{i=1}^{n}(\Delta t_{i})\times \exp\left[13.65-\frac{4000}{\frac{273+T(\Delta t_{i})}{T_{0}}}\right]$$

where

$\Delta t_{i}$ is the number of days where a temperature $T\;\left({}^{\circ}C\right)$ succeeds;
$T\left(\Delta t_{i}\right)$ is the temperature during the time period $\Delta t_{i}\cdot T_{0}=1\;{}^{\circ}C$.

Another equation was proposed for the prediction of both the development of CS and AGS with the time of hydration as follows [171,172]:

(19) $$L=\frac{x}{\frac{1}{m}+\frac{x}{n}}$$

where

*L* = predicted length change (i.e., CS, AS, DS or expansion);
*m* = initial rate of length change (IRL);
*x* = age (days);
*n* = ultimate length change (UL).

## 6. Conclusions and Recommendations

This paper provides a critical literature review consulting 172 references including 35 which were published during the last three years on chemical shrinkage (CS) and autogeneous shrinkage (AGS) of cementitious systems covering recent references with a small section on the effect of bio-fibers. Based on this review, several conclusions can be drawn as follows:

- Both shrinkage parameters behaved similarly in the first stage of hydration (at initial setting time). After that, the AGS seems to stabilize due to the hardening of the cementitious systems. However, CS continues to increase with the increase in hydration.
- CS development is a function of complex kinetic reactions for each mineral of binder in the system.
- The type of cement plays a role in controlling the CS and AGS of the cementitious system. Also, PC with high fineness particles increases the reaction rate rapidly, resulting in a further contraction phase of CSH gel and a reduction in the CS and AGS.
- Among SCMs, the most active supplementary compound that leads to minimize the autogenous shrinkage is the FA. However, the addition of slag with high content increases the AGS when compared to SF and FA. Moreover, incorporating calcium sulphate-based materials (CaSO_4_) in cement contributes to the retardation of hydration products which leads to a CS reduction. The incorporation of LF as a cement replacement accelerates the rate of chemical shrinkage.
- CS and AGS depend on the w/b ratio and are proportional to the degree of hydration, especially after 24 h of curing age. As the w/b ratio decreases, the CS and AGS increase. In addition, the greater the degree of hydration, the higher the CS and AGS.
- The addition of chemical admixtures can play a role in reducing CS and AGS of concrete. Adding 2% of SRA reduces the AGS by approximately 40% compared to the control mix.
- Using bio-fibers (PA) resulted in a significant reduction in the CS and AGS of cementitious composites. This reduction can range from 21.4% to 54% when 2% PA fibers were added.
- Future research could investigate the effect of different types of fibers including synthetic and bio-fiber on the CS and AGS in cement-based materials. Also, using fibers in geopolymer mixes can form an area for future study.
- More research is required regarding the impact of bio-fibers on the lifespan of cementitious composites. These may include different treatment methods and their effect on CS and AGS.

## Data Availability

Data sharing is not applicable to this article.

## Nomenclature

CS	Chemical shrinkage
AGS	Autogenous shrinkage
TCS	Total chemical shrinkage
ECS	External chemical shrinkage
P	Portland cement
HPC	High-performance concrete
UHPC	Ultra-high-performance concrete
SCMs	Supplementary cementitious materials
FA	Fly ash
SF	Silica fume
LF	Limestone fines
LVDT	Linear variable differential transducer
GBFS	Ground blast furnace slag
Mk	Metakoalin
FA-G	Fly-ash-gypsum
IH	Internal humidity
w/c	Water cement ratio (c is cement and w is water)
w/b	Water binder ratio (b consists of cement and SCM)
